# Supplementary material for: Copper Chaperone for Superoxide Dismutase FoCCS1 in Frankliniella occidentalis May Be Associated with Feeding Adaptation after Host Shifting
Source: Insects. 2022 Aug 29;13(9):782. doi: 10.3390/insects13090782 (PMC9501208; doi:10.3390/insects13090782)
Supplement: Supplementary file 1 [file insects-13-00782-s001.zip › Article_SupportingInformation.pdf]

Supporting Information

Additional supporting information may be found online in the Supporting Information section at the end of the article.

Table S1 Primer sequences used in CDS cloning

Primer name	Primer sequences (5' - 3')	Primer use
FoMnSOD2-F	ATCCTCTAAAATGCTGTCCCTTC	cDNA cloning
FoMnSOD2-R	TCCGAGAAACGCTTGGAGAC	
3′FoMnSOD2 Outer	GATTACGCCAAGCTTCTGGCAGAACCTTACTCCTGA	Amplification of 3'end cDNA(3'-RACE)
3′FoMnSOD2 Inner	GATTACGCCAAGCTTCCTCATCCCATTGCTGGGTA	
c FoMnSOD2-F	TTTGTTCGTTGTTTTGACCA	Verification of full-length cDNA
cFoMnSOD2-R	ACAAGTCTCGTTGAGTACAA	
FoCCS1-F	CGCAATTTGACGCAACTGGG	cDNA cloning
FoCCS1-R	ATCACCTTGTGTGGAATCGGT	
3′FoCCS1-Outer	GATTACGCCAAGCTTCACAGGCTGGCTTGTGGTAT	Amplification of 3'end cDNA(3'-RACE)
3′FoCCS1-Inner	GATTACGCCAAGCTTGGCTTGGGTGCAAGTGAAAA	
c FoCCS1-F	CAAGTTTGGTGGTGGTGTCAT	Verification of full-length cDNA
c FoCCS1-R	TCACCTTGTGTGGAATCGGTAT	

Table S2 Primer sequences used in quantitative reverse transcription PCR (RT-qPCR) and double-stranded (ds) RNA synthesis

Gene	Primer use	Sequence (5′-3′)	Product length
FoCCS1	qPCR	F：GGTTTAGTGCTGTTGGCGTC<br>R：CATGCAGCCCATGTTTACCAG	114 bp
FoMnSOD2	qPCR	F：TGCAAGGAACTACTGGCCTC<br>R：CCGAGAAACGCTTGGAGACA	147 bp
FoCCS1	dsRNA synthesis	F：TAATACGACTCACTATAGGG GATGCAATTCTCCGAGGTGT<br>R：TAATACGACTCACTATAGGG CAATACGAAAAACAGCCCGT	358 bp
	qPCR	F：GTGTGGGATATTATTGGCCG<br>R：ATTATACCACAAGCCAGCCT	122 bp
eGFP	dsRNA synthesis	F：TAATACGACTCACTATAGGG CCCTGAAGTTCATCTGCACC<br>R：TAATACGACTCACTATAGGG GTGCTCAGGTAGTGGTTGTC	481 bp
EF-1	qPCR	F: TCAAGGAACTGCGTCGTGGAT<br>R: ACAGGGGTGTAGCCGTTAGAG	130 bp

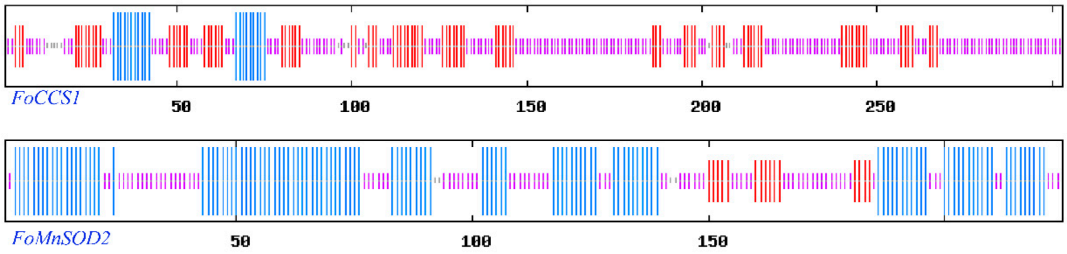

Figure S1: Secondary structure of the protein sequence of FoCCS1 and FoMnSOD2. Violet represents α-helices, blue represents random coils, and red represents extended strands.

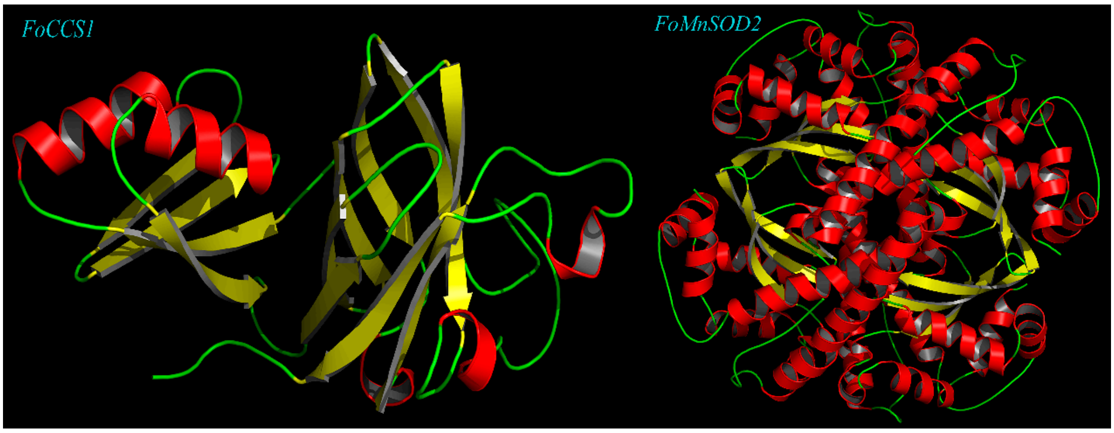

Figure S2 Predicted tertiary structure of FoCCS1 and FoMnSOD2 protein sequences. Red represents α-helices, yellow represents β-sheets, and green represents random coils.
