# Supplementary figures and images for: Copper Chaperone for Superoxide Dismutase FoCCS1 in Frankliniella occidentalis May Be Associated with Feeding Adaptation after Host Shifting
Source: Insects. 2022 Aug 29;13(9):782. doi: 10.3390/insects13090782 (PMC9501208; doi:10.3390/insects13090782)

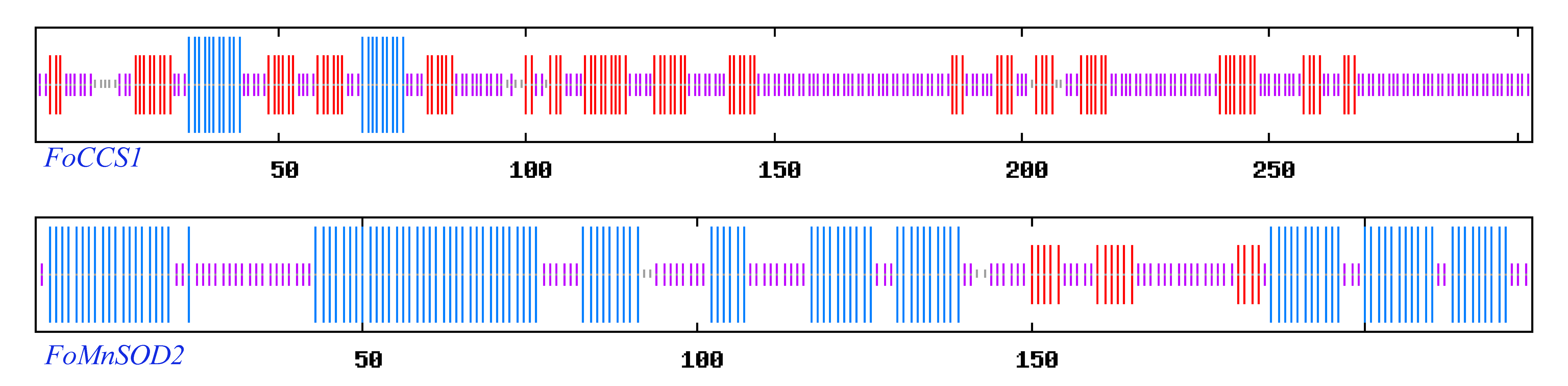

Supplement: Supplementary file 1 [file insects-13-00782-s001.zip › Figure S1.tif]
